# Health Effects of 12 Weeks of Team-Sport Training and Fitness Training in a Community Health Centre for Sedentary Men with Lifestyle Diseases

**DOI:** 10.1155/2018/1571807

**Published:** 2018-05-15

**Authors:** T. K. Møller, T.-T. Nielsen, R. Andersen, I. Lundager, H. F. Hansen, L. Ottesen, P. Krustrup, M. B. Randers

**Affiliations:** ^1^Department of Sports Science and Clinical Biomechanics, SDU Sport and Health Sciences Cluster (SHSC), University of Southern Denmark, Odense, Denmark; ^2^Department of Nutrition, Exercise and Sports, University of Copenhagen, Copenhagen, Denmark; ^3^University College Copenhagen, Copenhagen, Denmark; ^4^Prevention Centre Nørrebro, Copenhagen, Denmark; ^5^Sport and Health Sciences, University of Exeter, Exeter, UK

## Abstract

This study compares the effects of team-sport training, for sedentary men with lifestyle diseases, with fitness training in a pragmatic set-up in a community health centre (CHC). Thirty-two men in the fitness group (FiG) and 36 men in the team-sport group (TsG) completed the training and trained for 60–90 min, two times/week for 12–16 weeks. In FiG and TsG, mean heart rate (HR) during training was 73.2% and 74.5% of HR_max_, respectively. Percentage of training time above 90%HR_max_ was 6 ± 9% and 10 ± 15% and the percentage of participants who spent > 10% of total training time with HR > 90%HR_max_ was 20% and 41%, in FiG and TsG, respectively. In FiG, total fat mass was reduced by 3.5%  (*P* < 0.01), while performance in the 6 min walking test (6MWT) increased by 11%  (*P* < 0.001). In TsG, total fat mass was reduced by 2.2%  (*P* < 0.01), while 6MWT performance improved by 5%  (*P* < 0.05). Between-group differences were observed for systolic BP (*P* = 0.041) and mean arterial pressure (*P* = 0.050) in favour of TsG and for sit-to-stand test (*P* = 0.031) in favour of FiG. In conclusion, small-sided team sport is a worthy alternative to fitness training since the overall health effects are comparable, for example, improved balance and reduced fat mass. Team sport elicits high heart rates and improves cardiovascular health by reducing blood pressure, while fitness training improves sit-to-stand test performance related to activity of daily living.

## 1. Introduction

In Copenhagen, Denmark, citizens with, or at risk of developing, lifestyle diseases can be referred by their General Practitioner (GP) for physical activity in a community health centre (CHC). Considerable knowledge exists on the use of regular physical activity for the prevention and treatment of chronic diseases, specifically in relation to pathogenesis, diagnostic symptoms, physical fitness, and quality of life [1, 2]. Aging individuals regularly participating in training can improve and maintain their physical capacity and body composition and thereby their performance in tests related to activity of daily living [3, 4]. Traditionally, prevention and rehabilitation programmes have proposed brisk walking, running, cycling, and classic strength training as solutions. However, participants report difficulties in maintaining their training habits and healthier lifestyle after the supervised training period [5, 6]. To support the development of an active lifestyle and long-term continuance of physical activity, it is therefore important to find attractive activities that simultaneously promote health, support opportunities for continuing with a healthier lifestyle, and can be implemented in CHCs. To target various fitness capacities and their relationship to the risk of certain lifestyle diseases, it is important to include different training components that induce adaptations in different physiological systems. Recreational football encompasses endurance training, high-intensity interval training (HIIT), and strength training [7], and studies have shown broad-spectrum health effects after 12–16 weeks of football training, including improved maximum rate of oxygen consumption (VO_2max_), balance, fat oxidation, muscle mass and muscle strength, and lowered fat mass and fat percentage, blood pressure, and resting heart rate [4, 8–10]. The many intense acceleration instances, deceleration instances, and changes of direction stimulate musculoskeletal fitness [1, 11] and metabolic fitness, while the high heart rates (mean HR 81–89%HR_max_) stimulate the cardiovascular system [12]. The intermittent nature of recreational football organised as small-sided games with many brief intense actions is observed irrespective of age, gender, social status, and previous experience of the activity [13]. Moreover, it has been shown that team-sport, such as recreational football, can inspire long-term adherence to physical activity, as these activities are considered to be joyful and fun and support interparticipant relations, in contrast to the fitness training traditionally used in CHC set-ups [14–16].

In this study, we therefore sought to develop CHC-implementable team-sport activities with an activity profile comparable to that of recreational football, which were therefore expected to result in broad-spectrum health-promoting adaptations like those seen after 12–16 weeks of recreational football. This study also aimed to test the health effects of these activities in a pragmatic, real-life set-up, in contrast to the highly controlled studies previously conducted in laboratories, and to compare the effects of these team-sport activities on the fitness training normally used in CHCs for men with one or more lifestyle diseases, namely, hypertension, cardiovascular disease, Type II Diabetes, chronic obstructive pulmonary disease (COPD), and/or obesity.

## 2. Methods

### 2.1. Study Design and Recruitment Process

The design has a pragmatic, real-life approach to the daily context of work for the employees at the CHC. The physiotherapists at the CHC work in a setting with a heterogeneous group of participants and must conduct an in-process evaluation of the training including the physical abilities and disabilities of the participants, activity flow and intensity, and prior experiences with the activity pattern of fitness and team-sports. The Municipality of Copenhagen, the CHC, and the research group at Copenhagen University aimed to examine the physiological effects of the fitness training under the habitual standard procedures (see below). Furthermore, the aim was to examine whether it was possible to conduct team-sport training under these standard procedures and examine possible physiological effects of team-sport training. The standard procedures included the following: a continuous inclusion of citizens with a referral from their GP, the use of the employed physiotherapists as instructors, and all training conducted at or in relation to the CHC facilities. The CHC facilities were a gym of 10 × 10 meters in which primarily all training was conducted. The gym was equipped with machines for strength and cardiotraining during the fitness intervention and subsequently cleared for the team-sport intervention.

The study was carried out over a 2-year period. To study the effects of the fitness training normally offered in the CHC, participants for the fitness group (FiG) were recruited during the first year. After one year, all training sessions in the CHC were replaced with team-sport training (TsG) and the participants for this group were recruited during the second year.

All referred male citizens with lifestyle diseases (such as hypertension, dyslipidaemia, Type II Diabetes, or obesity) or at risk of developing these diseases were offered to participate in the study.

In a preliminary meeting, the employees at the CHC informed the citizens about the project and invited them to an information meeting with a representative of the scientific group. All citizens were informed that training was the same (year 1: fitness and year 2: team-sport training) for all citizens regardless of whether they participated in the study. Thus, participation in the study involved testing before and after the training period at the CHC. Citizens were allocated to training groups consisting of 12 citizens, and each group could consist of citizens participating in the study and those not participating. As a consequence of a continuous inclusion in the training group, all 12 citizens in a group did not start at the same time.

At the information meeting, all participants were informed of the risks associated with the experiment before giving their informed, written consent to participate. The study conformed to the Code of Ethics of the World Medical Association (Declaration of Helsinki) and was approved by the Ethics Committee of Copenhagen (H-1-2014-014).

**Figure 1:**
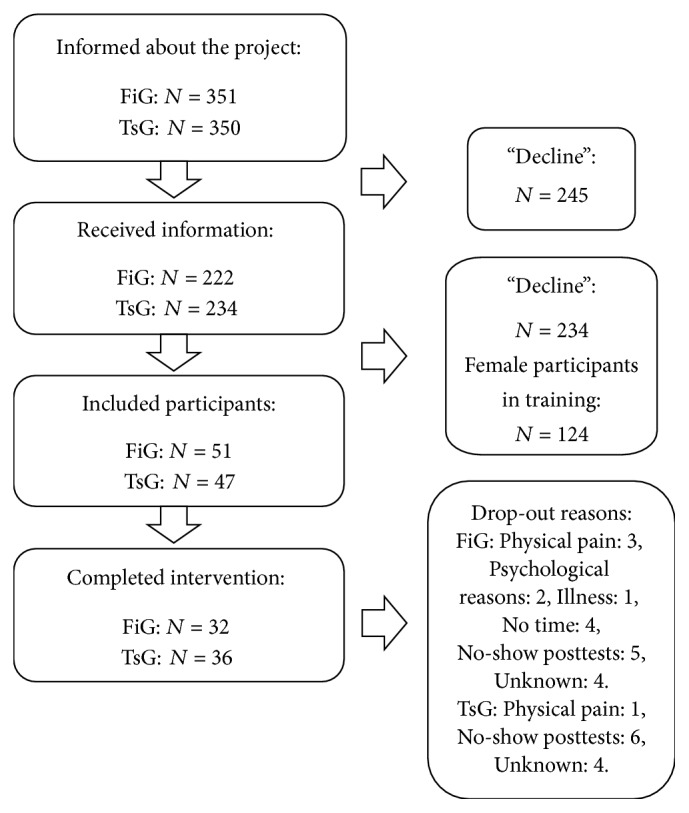
Recruitment process for all participants in year 1: fitness and year 2: team sport.

After the information meeting, 51 men were included in year one in FiG and 47 men in year two in TsG. The recruitment process is shown in Figure 1.

### 2.2. Participants

A total of 98 sedentary men gave their written informed consent to participate. There were no baseline differences between FiG and TsG, except for sit-to-stand (STS) performance (FiG; 12 ± 4, TsG; 14 ± 4, *P* = 0.027). Thirty men dropped out of the study: 19 from FiG and 11 from TsG (Figure 1). No between-group differences were found in baseline values and referral reasons in the 68 participants who completed the training (as per protocol, Table 1). Blood pressure values from participants with altered antihypertension drug use during the intervention period were removed from analysis (three participants from TsG; *N* = 33 in systolic blood pressure (SBP), diastolic blood pressure (DBP), and mean arterial pressure (MAP) and five participants from FiG; *N* = 27 in SBP, DBP, and MAP). Of the participants in FiG and TsG, 19 and 22 participants, respectively, were antihypertension drug users; 10 and 15 participants, respectively, were statin drug users; and 11 and 13 participants, respectively, were diabetic drug users.

In FiG, no between-group differences were found in baseline values between the participants who completed the training and drop-outs. In TsG, there were between-group differences in baseline values between participants who completed the training and drop-outs in respect of the following parameters: drop-outs had higher SBP (143 ± 19 versus 129 ± 15 mmHg, *P* = 0.015), higher MAP (103 ± 13 versus 95 ± 10 mmHg, *P* = 0.038), lower fat percentage (26.8 ± 12.6 versus 35.7 ± 7.3%, *P* = 0.006), and better STS performance (16 ± 6 versus 13 ± 3, *P* = 0.040).

### 2.3. Training

Physiotherapists employed at the CHC carried out the training in both groups. In the first year, the training consisted of fitness training and in the second-year activities were based on small-sided team-sports. At the end of the first year, the physiotherapists attended a workshop organised by scientific employees of the Copenhagen Centre for Team Sport and Health, University of Copenhagen, to define the content of team-sport training and educate the physiotherapists in conducting team-sport training. Based on this workshop, leading CHC staff developed a Team-Sport Manual with games and possible adjustments for use during training. Team-sport training sessions were conducted mostly indoors, and the games were adjusted to the facilities.

Each participant was offered 12 weeks of training, 2 × 90 min per week, except for the obese participants (BMI > 35) (FiG: *N* = 7; TsG: *N* = 5), who were offered training for 16 weeks, 2 × 60 min per week. Randomly selected training sessions for FiG and TsG were observed to examine the physical load during the two types of training. During the two-year project, a total of 55 training sessions were observed for description purposes (FiG: 21; TsG: 34) and 32 participants (FiG: *N* = 10; TsG: *N* = 22) had heart rate recorded during training. Moreover, rating of perceived exertion (RPE) was measured using visual analogue scale (VAS) scores for general RPE and specified for the legs and for respiration.

The fitness training included a light cycle or treadmill warm-up, aerobic training at 50–80% of estimated HR_max_, 6–10 individually assigned strength exercises in machines at 12–15 repetition maximum (RM) after an initial 1-2 weeks at 20 RM, and intensity drills at 70–80% of estimated HR_max_ (conducted as 4–8 intervals of 0.5–3 min). The team-sport training focused on one specific team-sport activity at a time and altered every fourth week. These activities were popular ball games (such as recreational soccer, team handball, basketball, volleyball, and floorball) adjusted to the participants. The training included warm-up exercises (adjusted to the team-sport activity of current use), technical exercises, one-a-side (1v1) exercises, and up to six-a-side (6v6) small-sided games of 3-4 min intervals with 2 min breaks in between and 5–7 repetitions. Common to both types of training were theories about physical exercise and healthy diet plus flexibility exercises. In both groups, the teams were mixed men and women, and the training was free of charge for the participants.

### 2.4. Measuring and Test Procedures

#### 2.4.1. Measurements during Training

Heart rate was recorded at 5 s intervals by short-range radio telemetry (Polar Team 2 System, Polar Electric Oy, Kempele, Finland), and data were transferred to a computer using the Polar Team 2 version 1.4.5 software (Polar Team2 System). Data are expressed in relation to individual maximal heart rate (HR_max_) and in HR zones < 70, 70–80, 80–90, and 90–100%HR_max_. Only complete HR recordings without technical fall-outs are included in the analysis. Individual HR_max_ was determined as the highest value reached either during an incremental cycle test to exhaustion or during training.

#### 2.4.2. Physiological Tests

The participants were instructed not to eat, drink, or smoke in the 2 hours prior to testing. With an empty bladder, whole-body and regional fat mass and lean body mass (LBM) were determined by DXA scans (LUNAR, GE Medical Systems, USA). After minimum 10 min of rest in a supine position in a quiet, dimly lit room, blood pressure was measured at least six times by an automatic upper-arm blood-pressure monitor (HBP-1300; OMRON, Illinois, USA) and an average value was calculated. Resting heart rate (RHR) was measured by OMRON HBP-1300 and Polar System and the lowest registered value was applied. Pulmonary gas exchange (Masterscreen CPX, CareFusion, Germany) was recorded at 5 s intervals during the incremental cycling test performed to exhaustion to determine maximal aerobic power (VO_2max_). The participants started exercising at a self-chosen pace of >50 rpm and a load of 40 W, and after 2 min the workload was increased by 10 W every 30 s until voluntary exhaustion. VO_2max_ was determined as the highest value achieved during a 30 s period. A respiratory exchange ratio (RER) of minimum 1.05 in combination with/or levelling off was set to determine the level of maximal oxygen uptake. All physiological tests were conducted at the University of Copenhagen by exercise physiologists.

#### 2.4.3. Performance Tests

On a separate day before and after the training period, the participants performed three standardised tests. The performance tests were conducted at the CHC by the physiotherapists, according to their standard procedure.

(1) A sit-to-stand (STS) test was performed to test the participants' ability to perform repeated standing up from a chair. Using a chair with a seat height of 45 cm above ground, the participants were instructed to sit in the middle of the chair with their back straight, their arms crossed over their chest and their feet flat on the floor. The correct technique was demonstrated to the participants, first slowly, then quickly. The participants practiced one to two repetitions before the start of the test. On the signal “go,” the participant rises to a full standing position, then returns to a seated position, and repeats this as many times as possible in 30 s.

(2) The participants performed a tandem balance test [17] involving four levels (duration 4 × 10 s), each resulting in 0–10 points (seconds). The four levels included standing up for 10 s with parallel feet, feet in semitandem position, full-tandem position with open eyes, and full-tandem position with closed eyes. The participants could achieve a maximum of 40 s. Participants who scored a total of 40 s in the baseline test were therefore removed from analysis.

(3) A 6 min walking test was performed. The participants walked for 6 min on a 25 m indoor track with a cone as a turning spot at each end. They were instructed to walk as fast as possible during the test. If the participant felt the need to take a break, they were instructed to do this standing up and, if necessary, supporting themselves against the wall.

### 2.5. Statistics

Baseline and follow-up data were tested for within-group effects with a paired-samples* t*-test. Between-group effects were tested with a univariate variance analysis using change scores as dependent value, baseline values as covariate, and group as factor. Between-group effect in intensity during training and VAS scores were compared with an independent-samples Mann–Whitney* U* test, and between-group differences in attendance were tested with one-way ANOVA. In FiG and TsG, respectively, baseline differences between participants who completed the training and drop-outs were tested with one-way ANOVA. All statistics were run in IBM SPSS statistics 24. A significance level of 0.05 was chosen. Data are presented as means ± SD or change scores (95% confidence interval (CI)). Nonparametric data (VAS, time spent in intensity zones, and relative heart rate) are presented as median (interquartile range (IQR)).

## 3. Results

### 3.1. Physiological Response to Training

**Figure 2:**
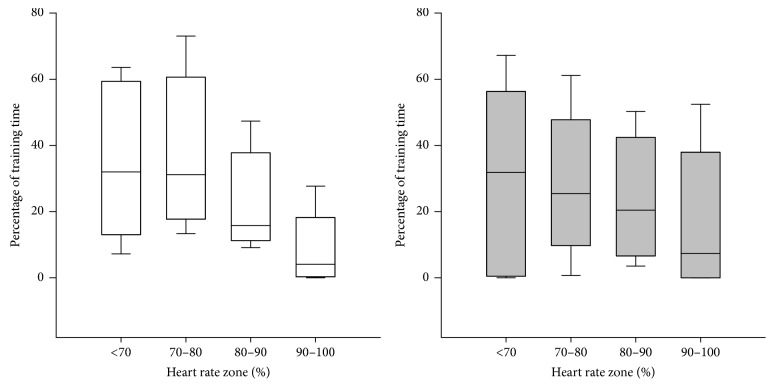
Training intensity - percentage of HR_max_. FiG: white; *N* = 10, TsG: grey; *N* = 22. Data are presented as median and 10th, 25th, 75th, and 90th percentile.

The relative heart rate during training was 73.2 (IQR: 70.1, 76.3) % of HR_max_ in FiG and 74.5 (IQR: 70.7, 78.4) % of HR_max_ in TsG, with no differences between groups (*P* = 0.604). 20% of the participants in FiG and 41% in TsG spent > 10% of total training time in heart rate zone > 90% of HR_max_. Average percentage of total training time above 90%HR_max_ was 5.8 ± 8.7% and 10.3 ± 14.6% for FiG and TsG, respectively. No between-group difference was observed in time spent in heart rate zones < 70%, 70–80%, 80–90%, and 90–100% of HR_max_ (Figure 2).

The participants rated general perceived exertion at 6.0 (IQR: 4.0–7.0) in FiG and 5.8 (IQR: 2.9–7.2) in TsG on a visual analogue scale (VAS). Specified values for the leg and respiration were 6.0 (IQR: 4.0–7.0) and 5.0 (IQR: 4.0–7.0), respectively, in FiG and 4.1 (IQR: 1.9–5.1) and 5.6 (IQR: 3.9–8.0) in TsG. There was a between-group effect for the specified perceived exertion for the legs (*P* = 0.025), with a lower perceived exertion in the legs in TsG than FiG.

#### 3.1.1. Training Attendance

There was no difference in attendance between FiG at 67 ± 28% and in TsG at 59 ± 24%, *P* = 0.209. This corresponds to an attendance of 1.3 and 1.2 times a week for FiG and TsG, respectively.

#### 3.1.2. Injuries during Training

No injuries were observed in FiG, and only one incident was observed in TsG, due to a participant suffering a minor pull in a lower leg muscle.

### 3.2. Blood Pressure

**Figure 3:**
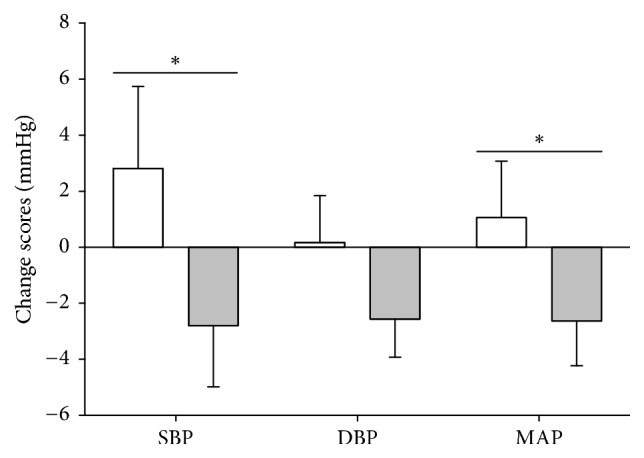
Changes in SBP, DBP, and MAP. FiG: white, TsG: grey. SBP: systolic blood pressure. DBP: diastolic blood pressure. MAP: mean arterial pressure. Data are presented as change scores ± SE. *∗* between-group effect in favour of TsG, *P* < 0.05.

In FiG, no change was found in SBP (132 ± 11 versus 134 ± 15 mmHg, *P* = 0.344), DBP (79 ± 8 versus 78 ± 8 mmHg, *P* = 0.922), or MAP (96 ± 8 versus 97 ± 10 mmHg; *P* = 0.309). In TsG, no change was found in SBP (128 ± 15 versus 125 ± 14 mmHg; *P* = 0.210), whereas DBP tended to be lowered (77 ± 8 versus 75 ± 9 mmHg; *P* = 0.068). Moreover, MAP was unchanged in TsG (94 ± 10 to 91 ± 10 mmHg; *P* = 0.108). Significant between-group differences were found in SBP (*P* = 0.041) as the change in TsG was −7.0 (CI: −13.9, −0.3) mmHg different from the change in FiG. In MAP the change (*P* = 0.050) in TsG was −4.6 (CI: −9.2, −0.0)  mmHg different from the change in FiG, whereas the change in DBP for TsG tended (*P* = 0.099) to be different by −3.3 (CI: −7.1, 0.6) mmHg compared to FiG. (Figure 3). There were no within-group or between-group differences for RHR.

### 3.3. Aerobic Fitness

Aerobic fitness tended (*P* = 0.051) to increase in FiG (21.7 ± 5.9 versus 23.5 ± 7.0 mlO_2_/min/kg) but was unchanged in TsG (Table 2). No between-group effects were observed for aerobic fitness (*P* = 0.662).

### 3.4. Body Composition

Total body mass decreased (*P* = 0.009) by 1.6% in FiG (105.4 ± 28.4 versus 103.5 ± 28.8 kg) and by 1.4% in TsG (99.7 ± 25.0 versus 98.4 ± 24.2 kg, *P* = 0.021). Fat mass decreased (*P* = 0.008) by 3.5% in FiG (39.1 ± 16.2 versus 37.6 ± 16.3 kg) and by 2.2% in TsG (36.8 ± 14.5 versus 35.9 ± 13.8 kg, *P* = 0.012). Fat percentage decreased (*P* = 0.039) by 2.3% in FiG (36.0 ± 7.3 versus 35.0 ± 7.4%) but was unchanged in TsG. The change in FiG was, however, not different from TsG as no between-group effects were observed (Table 2).

There were no within-group or between-group changes in lean body mass (LBM).

### 3.5. Functional Capacity

STS performance increased (*P* < 0.001) by 29% in FiG but was unchanged in TsG. The performance in the balance test and 6MWT increased in both groups (FiG; *P* = 0.048 and *P* < 0.001, for balance and 6MWT, resp., and TsG; *P* = 0.024 and *P* = 0.033, for balance and 6MWT, resp.). There was a between-group difference in STS performance (*P* = 0.031, Table 2).

## 4. Discussion

The main finding of this study was that traditional fitness training (FiG) and small-sided team-sport activities (TsG) in a CHC, within the standard procedures of the CHC, led to comparable effects on various health parameters with minor differences between training modalities. Positive effects on blood pressure were only found in TsG, whereas a reduction in fat mass, improvement in postural balance, and increased performance in a 6-minute walking test were found in both groups. Increases in sit-to-stand test performance were only found in FiG.

SBP and MAP were reduced in TsG by 2.8 and 2.6 mmHg, respectively, after the training period. According to the definition of hypertension as SBP > 140 mmHg and/or DBP > 90 mmHg [1] neither of the groups was hypertensive at baseline. However, cardiovascular mortality lowers in a log-linear relationship with a decrease in blood pressure to a SBP at 115 mmHg and a DBP at 75 mmHg [18]. Thus, cardiovascular health profile can be considered improved with a decrease in blood pressure to at least these values. The decreases in blood pressure seen in TsG are similar to average results seen in response to dynamic aerobic endurance training [19], but not as large as the reduction of 5–8 mmHg seen in studies with recreational football training [20, 21]. A study by Randers and colleagues [22] on the effects of street basketball found no changes in blood pressure after 12 weeks of training 3 versus 3 on one basket (10 × 12 m). The small playing area may cause more static muscle work compared to the dynamic muscle work during the more run-based activity profile during team sport on larger pitches, which may affect the training response. It was, moreover, surprising that no changes were found in blood pressure in FiG. In the present study, the participants were referred to training at the CHC by their GP and were a mix of individuals with one or more lifestyle diseases such as hypertension, obesity, high cholesterol, and Type II Diabetes. Both FiG and TsG were therefore quite heterogeneous, and participants with low baseline blood-pressure values lowered the average value. It may be suggested that the lower response to small-sided team sport, compared to recreational football, may in part be due to the low baseline values. However, blood pressure was unchanged in those participants with SBP and DBP above 140/90 mmHg at baseline (FiG; *N* = 9, TsG; *N* = 5). In the studies on recreational football, weekly training attendance was 2–2.5, which was higher than in the current study. One study has, however, shown that one weekly training session of recreational football can alter the blood pressure [23]. A low and maybe even a nonregular adherence could have influenced the training response. With a larger group of participants, it would have been interesting to analyse the pattern of attendance of the participants to the training sessions. Whether the pattern of physical activity influenced effects in blood pressure in the present study is therefore unknown. Nonetheless, a study by O'Donovan et al. (2017) showed that a training adherence below the recommended and a concentrated training with one-two weekly sessions may be sufficient to reduce the risk of all-cause mortality [17].

Aerobic fitness is another strong predictor of cardiovascular disease and early death [24]. In the present study, aerobic fitness was unchanged after TsG and tended (*P* = 0.051) to increase in FiG by 1.1 ml/min/kg. It was unexpected that TsG did not lead to improved aerobic fitness, as several studies using team-sport interventions have shown marked effects on aerobic fitness, with 3-4 mlO_2_/kg/min increases after 12–16 weeks of recreational football training for untrained healthy men and women [7, 25, 26] as well as men and women with hypertension or Type II Diabetes [20, 27]. In these studies, training intensity and weekly training volume were somewhat higher than in the present study, which may explain the lack of change in VO_2max_ in the present study.

The mean training intensity was similar in both groups, but 20% of the participants in FiG spent more than 10% of the total training time with heart rate above 90%HR_max_ compared to 41% of the participants in TsG. Average HR and time spent with HR above 90%HR_max_ are somewhat lower in TsG than seen in several intervention studies using comparable activities [4, 13, 21, 28, 29]. A key difference between the present study and these studies is the smaller pitch size of 10 × 10 m. Some studies on untrained individuals have shown physiological effects after small-sided activities with small pitch sizes (~10 × 10 m) [22, 30]. But a small pitch size limits the possibility of running with high speed, which may have caused the overall intensity to be lower than expected.

Although the mean exercise intensity was comparable between groups it is interesting that the participants in FiG rated a higher RPE for the legs than the participants in TsG. This is in keeping with several studies that found the perceived exertion in recreational football lower than in other types of training despite exercise intensity being similar or even higher during recreational football [8, 31]. The fact that the team-sport training in the present study was perceived to be easier than the fitness training may for some participants be a central element in the motivation to maintain an active lifestyle. A key element to maintain an active lifestyle is according to Nielsen and colleagues [15] the playful social interactions in team-sports as opposed to the individual nature of fitness training. The positive social relations and playful character of team sport can be sufficiently enjoyable to create a lower perceived exertion [31] and reason for continuation of a physical active life style [15].

The lower intensity during training in TsG combined with a low attendance rate in the training period reduced the overall training volume compared to other team-sport studies [4, 8, 28]. In a recent study on the effects of one weekly recreational football training session for 12 weeks, positive changes were observed in aerobic fitness and blood pressure [23]. Thus, it is likely that team-sport training can lead to several improvements in cardiovascular fitness with low volume if the intensity is high.

Training volume and energy expenditure are important for changing body weight and fat mass. In the present study, fat mass was lowered by 1.6 and 0.9 kg in FiG and TsG, respectively, with no differences between groups. This is in accordance with several reviews that found reductions of 1–3 kg of fat after approximately 3 months' training [19, 21, 32]. A decrease in fat mass may induce relevant health benefits, since development of Type II Diabetes and cardiovascular diseases is related to obesity [1]. We did not control the participant's diet, so we cannot conclude whether changes were due to training or changes in diet. A study comparing the effects of 12 weeks of diet or diet + football showed marked changes in body composition in both groups with no additional effect of playing football, but only the diet + football group improved lipid and lipoprotein profile [33].

Neither team-sport nor fitness training resulted in an increase in muscle mass in the present study. Other studies have shown a superior increase in either lean body mass after recreational football compared to continuous running at the same average intensity in men [7] or equal improvements between football and running in women [11]. However, a combination of the total volume in TsG, lower intensity, and pitch size in relation to number of participants may be related to the lack of development of muscle mass in this study.

Functional capacities improved in both groups, though muscle mass was unchanged. The increase in performance in STS was larger in FiG than TsG, which contrasts with a study that found similar changes in STS performance after recreational football and strength training [4]. Improvements in STS performance may be due to an increase in quadriceps muscle strength [34] and neuronal function [35], and these improvements in functional capacity indicate enhanced muscle endurance, coordination, and responsiveness, which is crucial for day-to-day activities and independence throughout old age.

## 5. Conclusion

In conclusion, this study provides new knowledge about the possibilities for using small-sided team-sport as the sole training method in community health centres. Team-sport training is a worthy alternative to the commonly used fitness training, as the overall health effect was comparable after team-sport training and fitness training. Minor differences were found between the two training modalities, with cardiovascular risk profile improving more after team-sport training than after fitness training and a greater improvement in sit-to-stand performance after fitness training than after team-sport training. The pragmatic, real-life design of this study was not able to show similar convincing effects on VO_2max_ and resting heart rate after small-sided team-sport activities as RCT intervention studies.

## 6. Perspectives and Practical Applications

To match the real-life setting of the CHC, the current study used a very applied approach. During year two of the project some key elements of consideration became clear. If the instructors had no experience or education related to team sport and ball games, the CHCs must consider supplementary education and prioritise practical sparring among the instructors. It is of great importance that the staff, who is to be instructors for small-sided team-sports, is focused on several aspects in the training sessions. To keep the intensity high on a 10 × 10 m pitch, the small-sided games should be organised as no more than 3-a-side. This will increase the area per participant, increase the number of actions for each participant, increase the possibilities for acceleration and direction changes, and simultaneously decrease the risk of impact between participants.

To maintain progression, motivation, and physiological development for each participant, the active role in the activities for each participant must be differentiated and changed according to the specific technical level of each participant. With extra focus on these aspects in the training sessions, the instructors can ensure a more appropriate adaption of the small-sided games to the specific group of participants. Furthermore, to enhance the possibilities of the participants to develop technical skills in collaboration, the CHC may consider using concomitant inclusion of participants, instead of a continuous inclusion.

## Figures and Tables

**Table 1:** Participants characteristics, per protocol.

	*N* FiG/TsG	FiG	TsG	Between-group differences *P* value
Age (year)	32/36	61 (11)	58 (14)	0.485
SBP (mmHg)	27/33	133 (13)	129 (15)	0.345
DBP (mmHg)	27/33	79 (8)	78 (8)	0.529
MAP (mmHg)	27/33	97 (9)	95 (10)	0.394
RHR (b.p.m.)	27/33	69 (8)	69 (11)	0.833
Weight (kg)	32/36	105.4 (28.4)	99.7 (25.0)	0.342
BMI (kg/m^2^)	32/36	33.9 (7.4)	32.3 (6.1)	0.360
LBM (kg)	32/36	61.7 (12.4)	58.5 (10.0)	0.239
Fat mass (kg)	32/36	39.1 (16.2)	36.8 (14.5)	0.526
Fat percentage (%)	32/36	36.0 (7.3)	35.7 (7.3)	0.847
Aerobic fitness (mlO_2_/min/kg)	22/29	22.4 (5.9)	21.7 (5.4)	0.659
*Referral (%)*	46/65			
Hypertension	11/13	23.9	20.0	0.980
Diabetes	9/15	19.6	23.1	0.294
BMI > 35	8/8	17.4	12.3	0.718
Dyslipidemia	6/12	13.0	18.5	0.207
Others	12/17	26.1	26.2	0.882

Baseline characteristics. Data are presented as means (SD). SBP: systolic blood pressure. DBP: diastolic blood pressure. MAP: mean arterial pressure. RHR: resting heart rate. LBM: lean body mass. High total referral count, because of multiple referrals for some participants.

**Table 2:** Effects of 12 weeks of traditional fitness training and team sport training.

	*N*, FiG/TsG	FiG Within-group	TsG Within-group	TsG estimated mean difference from FiG
Weight (kg)	32/36	−1.9 (−3.2, −0.5)^*∗*^	−1.3 (−2.4, −0.2)^*∗*^	0.5 (−1.2, 2.2)
Fat mass (kg)	32/36	−1.6 (−2.7, −0.4)^*∗*^	−0.9 (−1.5, −0.2)^*∗*^	0.6 (−0.6, 1.9)
LBM (kg)	32/36	−0.4 (−1.1, 0.2)	−0.2 (−0.8, 0.4)	0.2 (−0.7, 1.0)
Aerobic fitness (mlO_2_/kg/min)	22/29	1.1 (−0.0, 2.2)	0.5 (−0.5, 1.5)	−0.6 (−2.1, 0.8)
STS (stand/30 s)	19/15	3.5 (2.6, 4.5)^*∗*^	1.5 (−0.3, 3.3)	−2.1 (−3.9, −0.2)^*∗∗*^
Balance (s)	12/11	4.5 (0.0, 9.5)^*∗*^	3.6 (0.6, 6.5)^*∗*^	0.2 (−4.3, 4.7)
6MWT (m)	27/17	54.4 (33.3, 75.6)^*∗*^	27.2 (2.4, 52.0)^*∗*^	−28.0 (−57.7, 1.6)
Attendance (%)	29/34	67.4 (56.7, 78.2)	59.1 (50.7, 67.4)	−8.4 (−21.5, 4.8)^*∗∗*^

^*∗*^Within-group effects (*P* ≤ 0.05). ^*∗∗*^Between-group effects (*P* ≤ 0.05). Within-group data presented as change scores (95% CI), attendance as mean (95% CI), and between-group data as estimated mean difference (95% CI) with FiG as reference group. LBM: lean body mass. STS: sit-to-stand. 6MWT: 6-minute walking test.
